# A Benzoic Acid Derivative and Flavokawains from *Piper* species as Schistosomiasis Vector Controls

**DOI:** 10.3390/molecules19045205

**Published:** 2014-04-23

**Authors:** Ludmila N. Rapado, Giovana C. Freitas, Adriano Polpo, Maritza Rojas-Cardozo, Javier V. Rincón, Marcus T. Scotti, Massuo J. Kato, Eliana Nakano, Lydia F. Yamaguchi

**Affiliations:** 1 Laboratório de Parasitologia, Instituto Butantan, Av. Vital Brasil, 1500, São Paulo, SP, CEP 05503-900, Brazil; E-Mail: eliananakano@butantan.gov.br; 2 Instituto de Ciências Biomédicas, Universidade de São Paulo, Av. Prof. Lineu Prestes, 1374, São Paulo, SP, CEP 05508-000, Brazil; 3 Research Support Center in Diversity of Natural Products, Instituto de Química, Universidade de São Paulo, Av. Prof. Lineu Prestes, 748, sala 1124, São Paulo, SP, CEP 05508-000, Brazil; E-Mails: giovanacf@gmail.com (G.C.F.); majokato@iq.usp.br (M.J.K.); 4 Departamento de Estatística, Centro de Ciências Exatas e de Tecnologia, Universidade Federal de São Carlos, Via Washington Luís, km 235, Sao Carlos, SP, Caixa-postal 676, CEP 13565-905, Brazil; E-Mail: polpo@ufscar.br; 5 Department of Pharmacy, Faculty of Sciences, Universidad Nacional de Colombia, Kr 30 45-03, Bogotá, Colombia; E-Mails: marojasc@unal.edu.co (M.R.-C.); jrinconv@unal.edu.co (J.V.R.); 6 Centro de Ciências Aplicadas e Educação, Universidade Federal da Paraíba, Campus IV, Rua da Mangueira, s/n, Rio Tinto, PB, CEP 5829-7000, Brazil; E-Mail: mtscotti@gmail.com

**Keywords:** schistosomiasis, molluscicide, benzoic acid, flavokawain, *Piper*

## Abstract

The search of alternative compounds to control tropical diseases such as schistosomiasis has pointed to secondary metabolites derived from natural sources. *Piper* species are candidates in strategies to control the transmission of schistosomiasis due to their production of molluscicidal compounds. A new benzoic acid derivative and three flavokawains from *Piper diospyrifolium*, *P. cumanense* and *P. gaudichaudianum* displayed significant activities against *Biomphalaria glabrata* snails. Additionally, “*in silico*” studies were performed using docking assays and Molecular Interaction Fields to evaluate the physical-chemical differences among the compounds in order to characterize the observed activities of the test compounds against *Biomphalaria glabrata* snails.

## 1. Introduction

Parasitic diseases are a major public health problem, especially in the developing countries. Schistosomiasis is considered by the World Health Organization (WHO) to be the second most relevant tropical disease [1]. The prevalence of this malady results primarily from a lack of basic sanitation. Human schistosomiasis transmission begins with the penetration of the skin by larval cercariae living in fresh water. Subsequently, the parasite continues development in multiple human organs [2,3]. Progressive efforts have been made to eradicate the disease, and one of the key strategies to control of the intermediate host, snails of the *Biomphalaria* genus, which act as a transmission vector of cercariae [4]. Currently, a commercially available niclosamide-based molluscicide, Bayluscide^®^ (Bayer) is the most effective compound employed in schistosomiasis control programs [5]. However, there are several negative aspects of Bayluscide^®^ use, including compound resistance and toxicity to other organisms [6].

The continued importance of natural products as a source of new lead compounds has led to the search for molluscicidal compounds in the plethora of plants found in tropical forests [7]. The effective molluscicidal activity of crude extracts derived from the *Piper* species supported further investigation of alternative compounds to control snail populations [8]. *Piper diospyrifolium* crude leaf extract demonstrated high activity against *Biomphalaria glabrata.* Thus, the phytochemical study of leaf extract resulted in the isolation and structural determination of a new active benzoic acid derivative. Additionally, flavokawain A was isolated from crude leaf extracts and its activity was compared to 2',4',6'-trihydroxydihydrochalcone and dihydroflavokawain C isolated from *P. cumanense* and *P. gaudichaudianum*, respectively. A comparison of benzoic acid derivative activity was made with commercially available analogs, hydroquinone and *p*-hydroxybenzoic acid. 

Additionally, “*in silico*” studies were performed by docking analysis using a RXR-like protein (retinoid X receptor) as a potential drug target for *B. glabrata*. RXR-like proteins belong to the nuclear receptor (NR) protein family, a superfamily of transcription factors present in metazoans [9], which regulate various biological processes including cell growth, development and metamorphosis [9]. The capacity of these nuclear receptors to bind small molecules might be used to control several biological processes [9,10]. The receptor-mediated effects are stimulated and/or inhibited by endogenous cognate ligands specific for each NR, but also by exogenous substances including natural products and synthetic chemicals. Considering these promising pharmacological targets, NRs and their ligands have attracted scientiﬁc interest, particularly for drug discovery and in toxicology and environmental science [10,11].

Molecular interaction fields were also employed to characterize the similarity and physical-chemical differences among the test compounds in order to explain the differences in activity profiles against *B. glabrata* [12].

## 2. Results and Discussion

### 2.1. Compound Purification

Fractionation of *P. diospyrifolium* leaf extracts was performed using flash purification leading to the isolation of flavokawain A **1** and the new benzoic acid derivative **2**. Purified compound **1** was obtained as yellow crystals. The molecular mass was determined as C_18_H_18_O_5_, based on HRESIMS data which displayed an [M+H]^+^ quasi-molecular ion peak at *m/z* 315.1231 (calculated for C_18_H_18_O_5_ [M+H]^+^ = 315.1227). Comparison of its spectral data with literature values identified compound **1** as flavokawain A [13]. 

Compound **2** was a dark yellow solid. The HRESIMS analysis indicated the presence of a quasi-molecular ion peak at *m/z* 357.2060, corresponding to the formula C_22_H_28_O_4_ (calculated for C_22_H_28_O_4_ [M+H]^+^ = 357.2060). The ^1^H-NMR spectral data (Table 1) indicated the presence of one 1,3,4-trisubstituted aromatic ring according to the signals at δ 8.59 (d, *J* = 2.0), 7.03 (d, *J* = 10.0) and 8.16 (dd, *J* = 10.0, 2.0) assigned to H2, H5 and H6, respectively (Table 1). Singlet signals at δ1.65 (3H), 2.26 (3H), 1.65 (3H) and 1.57 (3H) were assigned to methyl groups linked to sp^2^ carbon atoms. The farnesyl side chain was determined through the association of these methyl groups with triplet signals at δ 6.87, 5.17 and 5.09 of the olefinic hydrogens.

**Figure 1:**
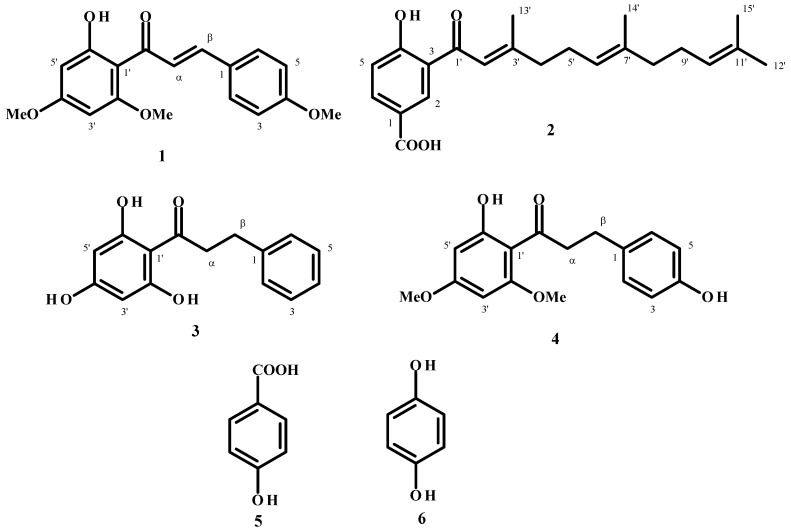
Chemical structures of the compounds assessed in this study.

The ^13^C-NMR spectral data indicated the presence of one carboxyl group of a benzoic acid at δ 170.90. In addition, six aromatic carbon signals were detected in the range of δ 119 to 168, assigned the 3-alkyl-4-hydroxybenzoic acid moiety. The remaining signals were attributed to a farnesyl moiety with six of them δ 118.80, 164.10, 127.70, 136.82, 124.30 and 131.57 corresponding to three double bonds, δ 42.11, 26.81, 39.79, 26.30 were assigned to methylene groups and δ 25.91, 20.57, 16.28 and 17.80 to methyl groups of the farnesyl group (Table 1). A comparison of data in the literature [14] with that of compound **2** corroborated the structure this compound as 4-hydroxy-3-(3,7,11-trimethyldodeca-2,5,10-trienyl)benzoic acid (Figure 1).

Compound **3**, a yellow crystalline substance, was isolated from *P. cumanense* leaves after extensive purification steps based on silica gel chromatography. The HRESIMS data determined its molecular formula to be as C_15_H_14_O_4_ (*m/z*: 259.0980 [M+H]^+^ obtained, calculated [M+H]^+^ = 259.0970). Comparison of NMR data with the literature identified this compound as 2',4',6'-trihydroxydihydrochalcone [15].

Compound **4** was isolated from leaves of *P. gaudichaudianum* and had a quasi-molecular ion peak at *m/z*: 303.1236 [M+H]^+^ assigned to the formula C_17_H_18_O_5_, based on HRESIMS data (calculated for C_17_H_18_O_5_ [M+H]^+^ = 303.1232). NMR data and literature comparison identified this compound as dihydroflavokawain C [16].

**Table 1:** ^1^H (200 MHz) and ^13^C (50 MHz) NMR data of compound **2**.

Compound 2		
Position	^1^H (*J* Hz)	^13^C
1		120.31
2	8.59 (1H, d, 2.0)	133.16
3		119.70
4		167.90
5	7.03 (1H, d, 10.0)	119.01
6	8.16 (1H, dd, 10.0, 2.0)	137.17
1'		195.63
2'	6.87 (1H, s)	118.80
3'		164.10
4'	2.37 (2H, m)	42.11
5'	2.04 (2H, m)	26.81
6'	5.17 (1H, m)	127.70
7'		136.82
8'	2.37 (2H, m)	39.79
9'	2.04 (2H, m)	26.30
10'	5.09 (1H, m)	124.30
11'		131.57
12'	1.65 (3H, s)	25.91
13'	2.26 (3H, s)	20.57
14'	1.65 (3H, s)	16.28
15'	1.57 (3H, s)	17.80
COOH	13.49 (1H, s)	170.90

### 2.2. Molluscicidal and Ovicidal Activities

The molluscicidal and ovicidal activities of compounds **1** and **2** were evaluated against *B. glabrata*. The molluscicidal and ovicidal activities of commercially available analogs of the benzoic acid derivative, hydroquinone and *p*-hydroxybenzoic acid, and the two chalcones **3** and **4** isolated from *P. cumanense* and *P. gaudichaudianum*, were also evaluated (Table 2, Supplementary Table S1). Compound **2** was potent against adult snails among the natural products tested (LC_50_ 7.28 µg/mL) (Table 2). A concentration of 12 µg/mL resulted in 100% dead animals after 24 h of exposure (Supplementary Table S1). This benzoic acid derivative was significantly more potent compared with *p*‑hydroxybenzoic acid (LC_50_ 1302.91 µg/mL). The C_15_ isoprenoid side chain of **2** that confers lipophilicity to the compound can attach or insert in the mollusk membrane and may explain this difference in activity. Overall, a simple quinone **6** was the most active (LC_50_ 3.15 µg/mL) among the tested compounds. The quinone was also active against the embryonic stages of *B. glabrata*, however, the benzoic acid and compound **1** did not have any activity against these stages (Table 2) (Supplementary Table S1). A concentration of 9 µg/mL hydroquinone resulted in 100% dead adult animals after 24 h of exposure. In the embryonic stages, the blastula and gastrula stages were more sensitive than the trocophore and veliger stages with 100% dead animals after 24 h using 2, 4, 8 and 8 µg/mL hydroquinone, respectively.

**Table 2:** LC_50_ and LC_90_ (µg/mL) for *B. glabrata* in different developmental stages exposed to isolated compounds.

Compounds		Adults	Developmental stages			
			Blastula	Gastula	Trocophore	Veliger
Flavokawain A (**1**)	**LC_50_**	21.85 [19.22–24.21]	nc	nc	nc	nc
	**LC_90_**	27.97 [27.97–33.95]	nc	nc	nc	nc
4-Hydroxy-3-(3,7,11-trimethyldodeca-2,5,10-trienyl) benzoic acid (**2**)	**LC_50_**	7.28 [6.54–7.96]	nc	nc	nc	nc
	**LC_90_**	10.04 [10.04–11.76]	nc	nc	nc	nc
2',4',6'-Trihydroxy-dihydrochalcone (**3**)	**LC_50_**	5.35 [4.28–6.30]	10.18 [9.68–10.69]	10.31 [9.86–10.79]	10.71 [10.24–11.16]	11.83 [11.35–12.31]
	**LC_90_**	6.47 [6.47–9.06]	14.12 [14.12–15.90]	13.54 [13.54–15.28]	14.44 [14.44–16.23]	14.87 [14.87–16.19]
Dihydroflavokawain C (**4**)	**LC_50_**	nc	nc	nc	nc	nc
	**LC_90_**	nc	nc	nc	nc	nc
*p*-Hydroxybenzoic acid (**5**)	**LC_50_**	nc	nc	nc	nc	nc
	**LC_90_**	nc	nc	nc	nc	nc
Hydroquinone (**6**)	**LC_50_**	3.12 [2.70–3.59]	1.17 [1.14–1.20]	1.98 [1.92–2.04]	2.74 [2.60–2.89]	4.26 [4.12–4.41]
	**LC_90_**	5.27 [5.27–6.97]	1.51 [1.51–1.59]	2.86 [2.86–3.05]	5.83 [5.83–6.45]	6.17 [6.17–6.54]

nc = not calculated. [ ] 95% confidence interval. *n* = 30 snails for the adult stage. Values were obtained at the end of the 7th day of observation.

Three compounds with chalcone skeleton, **1**, **3** and **4**, were tested against *B. glabrata*. Flavokawain A (**1**) showed moderate molluscicidal activity (LC_50_ 21.85 µg/mL) and did not exhibit any activity in the embryogenic stages of the snail. Dihydroflavokawain C (**4**) did not exhibit any activity at any of the life stages of the snail. The compound with the most potent activity was 2',4',6'-trihydroxy-dihydrochalcone (**3**), which was active against all life stages of the snail. This compound was more active in the adults (LC_50_ 5.35 µg/mL) (Table 2, Supplementary Table S1), but in the embryonic stages caused a delay in development; after 7 days all the embryos had not completed development and remained in the eggs. The eggs were examined daily and regardless of heart beat detection did not emerge from the eggs. All of the embryos were deceased after 16 days.

**Figure 2:**
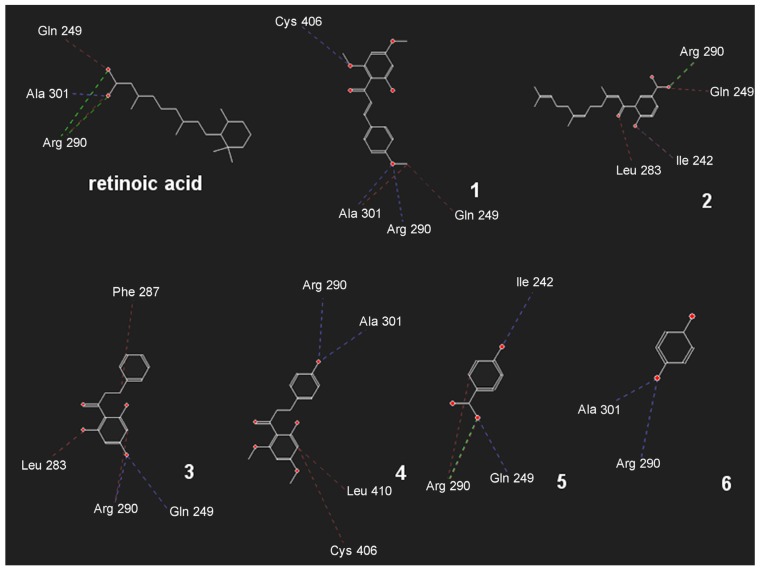
Best docking results showing electrostatic (green), hydrogen bonds (blue) and hydrophobic (red) interactions between the ligands and active site of RXR-protein like of *Biomphalaria glabrata* (PDB code: 1XIU).

Considering that RXR-like protein can regulate biological processes in *B. glabrata*, theoretical studies were performed, comparing the activity of the compounds and their binding energies in the active site looking for an antagonist compound for this protein. *In-silico* docking analysis determined that all of the compounds interact with the RXR-like protein at arginine 290 through hydrogen bonding (compounds **1**, **3**, **4** and **6**) or by electrostatic interaction in the benzoic acid derivatives (compounds **2** and **5**) in the same manner as the carboxyl group of retinoic acid (Figure 2). 

The analysis of the hydrophobic surface of the binding pocket site of the enzyme (Supplementary Figure S1) revealed that only small lipophilic compounds might bind in this site. Considering these observations, compound **2** and retinoic acid, each having a carboxyl group and a lipophilic side chain, are able to form electrostatic interactions with arginine 290 of the enzyme and insert the rest of the chain in to the hydrophobic region of the binding pocket. Compound **5** forms the same electrostatic interaction of compound **2** with arginine 290 and forms hydrogen bonds with glutamine 249 and isoleucine 242. Additionally, compound **3** forms hydrogen bonds with both residues. Compounds **1**, **4** and **6**, similarly to retinoic acid, form hydrogen bonds with arginine 290 and alanine 301 in the binding pocket site (Figure 2).

**Figure 3:**
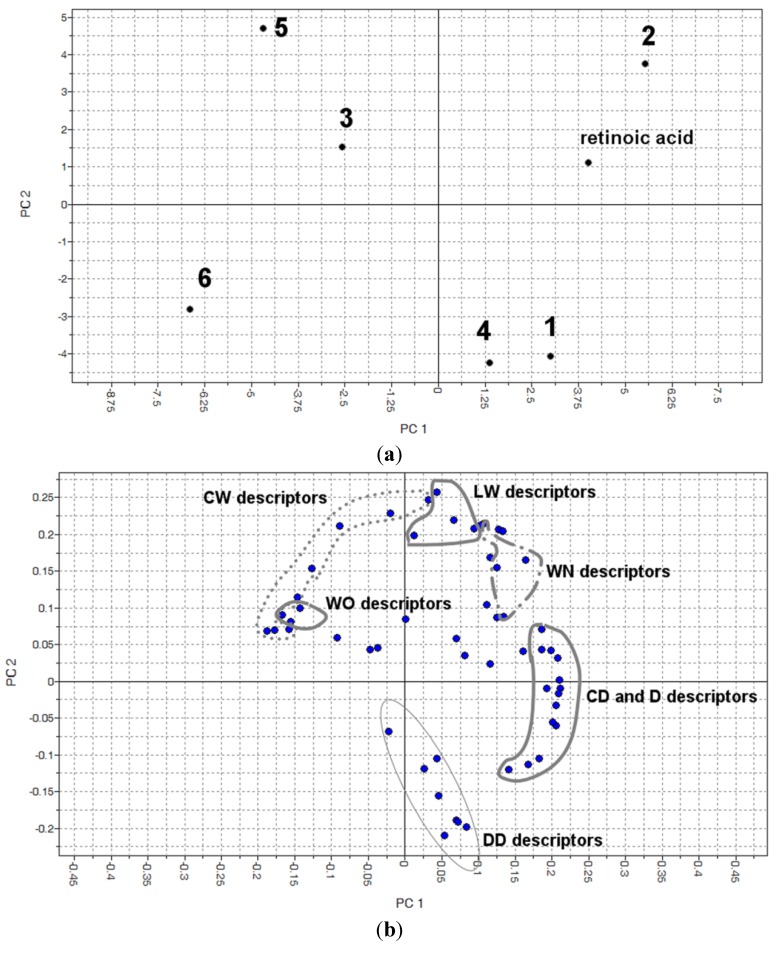
Scores (**a**) and loadings plot (**b**) of PCA generated using 57 descriptors generated by molecular interaction fields for probes water, amide nitrogen, carboxylic oxygen and DRY (lipophilic).

A PCA analysis was performed using the descriptors generated by the Molecular Interaction Fields (MIF) describing the similarity/dissimilarity of compound interactions with the lipophilic, water, hydrogen acceptor and hydrogen donor probes that represent the same interactions as the biological environment that these compounds were subjected to during the assay. The scores plot of PC1 and PC2 is presented in Figure 3 that explains 34.5% and 24.9% of the total observed variance. The loadings plot showed that lipophilic capacity factors descriptors (CD—hydrophobic volume per surface unit) and D descriptors (hydrophobic volumes generated by the lipophilic fields) were on the right side (positive contribution to PC1). Retinoic acid and compound **2** are located in the first quadrant, in the same position as the descriptors IW (Integy moment) and WN (Hydrogen bond acceptor volumes), which first encodes the unbalance between the center of mass of a molecule and the barycenter of its hydrophilic regions and second encodes the hydrogen bonding ligand acceptor fields, that contribute positively to PC1 and PC2 (Supplementary Figures S1 and S2). Compound **3**, located in the second quadrant, displays well-defined polar and lipophilic regions (Supplementary Figure S2). Compounds **5** and **6** were the smallest and more hydrophilic compounds and are located in the second and third quadrants, respectively. Their presence in these positions may be explained by the presence of descriptors CW (hydrophilic capacity factors/hydrophilic volume per surface unit) that confers a negative contribution to PC1, which represents the hydrophilic volume per surface unit of the molecule (Figure 3 and Supplementary Figure S2) [14]. Comparing compounds **1** and **4**, that are located in the fourth quadrant of the score plot, where DD (differences of the hydrophobic volumes) descriptors are common to both and encode the variation of hydrophobic volumes according the three dimensional conformation of the less active ligands [14]. 

GRID descriptors generated by MIF were evaluated to elucidate some of the similarities and dissimilarities between the compounds in order to characterize intermolecular interactions. The docking results showed that all compounds could interact in the same binding pocket as retinoic acid and the distribution of hydrophilic and hydrophobic regions corroborate with the analysis of the Volsurf approach. 

The pattern obtained in PCA permitted to identify the physical-chemical features of the tested compounds, such as the hydroxybenzoic acid or hydroxylated phenyl fragments (hydrophilic regions) with lipophilic chains (alkyl or aromatic), which were responsible for higher activity of compounds **2** and **3**, and consequently, accounted for the lower activity of compounds **1** and **4** which are abundant in hydrophilic regions. These structural features may be used as a guide for further synthetic optimization or to select new structures with potential activity. However, the physical-chemical differences of compounds **5**, **3** and **6** are not so simple to interpret to explain their activity and the inactivity of *p*-hydroxybenzoic acid.

## 3. Experimental Section

### 3.1. General

The ^1^H and ^13^C-NMR were recorded on a Bruker DRX500 (Billerica, MA, USA) spectrometer (200 MHz for ^1^H and 50 MHz for ^13^C) in CDCl_3_ using TMS as internal standard. HREIMS analysis was recorded on Bruker MicrOTOFQ-II (Bremen, Germany). HPLC analyses were performed in a Shimadzu (Kyoto, Japan) system with binary pumps LC-20 AD equipped with a UV detector SPD-20 A, column oven CTO 20A, control unit CBM20A, LCSolution for chromatogram manipulation and Phenomenex Luna 2.5 μm C18(2)-HST, 100 × 2 mm (Torrance, CA, USA), column. The mobile phase consisted of acetonitrile:water (0.1% formic acid) and flow rate of 0.2 mL/min. The column purification was performed using Biotage Flash (IsoleraOne) system (Uppsala, Sweden). The binary mobile phase consisted of hexane and acetyl acetate, the flow rate was kept in 12 mL/min for a total run time of 7 min. The system was run in a gradient mode: eight volumes of 17 mL of mobile phase (6% of ethyl acetate and 94% of hexane) and three volumes of 17 mL (26% of ethyl acetate and 74% of hexane). The peaks were monitored at 280 and 254 nm and the collected fractions were analyzed by HPLC.

### 3.2. Plant Material

*Piper diospyrifolium* Kunth. and *P. gaudichaudianum* Kunth. were collected in the garden of Chemistry Institute, University of São Paulo, Brazil. The voucher specimens K-431 and K-031 were deposited in the Herbarium at Instituto de Pesquisas Jardim Botânico do Rio de Janeiro and Prof. Elsie Franklin Guimarães from the same Institute identified the species. *Piper cumanense* H.B.K. was collected in Zapatoca, province of Santander, Colombia and identified by Prof. Ricardo Callejas (Universidad de Antioquia, Colombia). The voucher specimen (COL 468660) was deposited at the Herbarium of Universidad Nacional de Colombia.

### 3.3. Extraction and Isolation

Leaves of *P. diospyrifolium* (250 g) and *P. gaudichaudianum* (100 g) were dried in a 40 °C oven, milled and extracted 3× with MeOH (1 L) at room temperature. The extracts were filtered, and the organic phase was evaporated using a rotary evaporator. The crude extract (33 g, *P. diospyrifolium*; 18 g, *P. gaudichaudianum*) were dissolved in 10% of water in MeOH and filtered through Celite^®^ (Merck, Whitehouse Station, NJ, USA) and extracted with EtOAc, yielding 10 g and 6 g of organic fractions, respectively. Two hundred milligrams of each fraction (*P. diospyrifolium*, PD and *P. gaudichaudianum*, PG) was subjected to column chromatography using a Biotage system. The PD resulted in 22 fractions and analyses of fraction 16 and 21 resulted in the purification of the compounds **2** and **1**, respectively. The compound **4** was isolated from fraction 12 after PG fractionation in the Biotage. The compound **3** was isolated from *P. cumanense* leaves (1.5 kg) extracted with 95% of EtOH in water yielding 0.4 kg of crude extract (PC). The crude extract was partitioned with CH_2_Cl_2_ (PCDCM), MeOH (PCMeOH) and *n*-BuOH (PCBuOH). The PCMeOH phase (5 g) was subjected to a column chromatography using gradient of CH_3_Cl_3_ and EtOAc. The PCMeOH_19 fraction (2.5 g) was chromatographed on silica gel eluted with CHCl_3_–EtOAc (90:10) to EtOAc 100% to give 23 subfractions. Subfraction 6 (PCMeOH_19_sub6, 530 mg) was chromatographed by a silica gel column eluted with CHCl_3_–EtOAc (7:3) to give 6 subfractions. The subfraction obtained from this last separation (PCMeOH_19_sub6_2, 110 mg) was purified using column chromatography CHCl_3_–EtOAc (4:1) to obtain afford **3** (34 mg) as a white crystalline solid.

### 3.4. Spectral Data

*Flavokawain A* (**1**). Yellow crystals, UV (MeOH): 364, 252 nm. ^1^H-NMR (CDCl_3_) δ 7.57 (2H, d, *J* = 2.0, 6.0 Hz, H2 and H6), 6.93 (2H, d, *J* = 2.0, 6.0 Hz, H3 and H5), 5.96 (1H, d, *J* = 2.0 Hz, H3'), 6.11 (1H, d, H5'), 7.80 (1H, s, Hα and Hβ), 3.95 (3H, s, OMe-4), 3.84 (3H, s, OMe-2'), 3.86 (3H, s, OMe-4').^13^C-NMR (CDCl_3_) δ 127.77 (C1), 130.15 (C2), 114.38 (C3), 161.38 (C4), 114.38 (C5), 130.15 (C6), 127.82 (C1'), 166.05 (C2'), 91.26 (C3'), 162.48 (C4'), 93.82 (C5'), 168.37 (C6'), 142.54 (Cα), 125.12 (Cβ), 55.86 (OMe-4), 55.42 (OMe-2'), 55.87 (OMe-4'), 192.63 (C=O).

*4-Hydroxy-3-(3,7,11-trimethyldodeca-2,6,10-trienil) benzoic acid* (**2**). Dark yellow solid, UV (MeOH): 330, 254 nm. HR-ESI-MS *m/z*: [M+H]^+^for C_22_H_28_O_4_ = 357.2060; ^1^H-NMR (CDCl_3_) δ 8.59 (1H, d, *J* = 2.0 Hz, H2), 7.03 (1H, d, *J* = 10.0 Hz, H5), 8.16 (1H, dd, *J* = 10.0, 2.0 Hz, H6), 6.87 (1H, s, H3'), 2.37 (2H, m, H4'), 2.04 (2H, m, H5'), 5.17 (1H, m, H6'), 2.37 (2H, m, H8'), 2.04 (2H, m, H9'), 5.09 (1H, m, H10'), 1.65 (3H, s, H12'), 2.26 (3H, s, H13'), 1.57 (3H, s, H15'), 13.49 (1H, s, COOH). ^13^C-NMR (CDCl_3_) δ 120.31 (C1), 133.16 (C2), 119.70 (C3), 167.90 (C4), 119.01 (C5), 137.17 (C6), 195.63 (C1'), 118.80 (C2'), 164.10 (C3'), 42.11 (C4'), 26.81 (C5'), 127.70 (C6'), 136.82 (C7'), 39.79 (C8'), 26.30 (C9'), 124.30 (C10'), 131.57 (C11'), 25.91 (C12'), 20.57 (C13'), 16.28 (C14'), 17.80 (C15'), 170.90 (COOH).

*2',4',6'-Trihydroxydihydrochalcone* (**3**). Crystalline needles, UV λ_max_ (MeOH) nm: 324, 285. IR ν_max_ (KBr) cm^−1^: 3443, 2957, 1649, 1595, 1454, 1395, 1016. HR-ESI-MS *m/z*: 259.0980 [M+H]^+^for C_15_H_14_O_4_. ^1^H-NMR (CDCl_3_) δ 7.27 (4H, m, H2, H3, H5 and H6), 7.17 (1H, m, H4), 5.85 (2H, s, H3'), 5.85 (2H, s, H5'), 3.33 (2H, t, *J* = 7.8 Hz, Hα), 2.98 (2H, t, *J* = 7.8 Hz, Hβ). ^13^C-NMR (CDCl_3_) δ 143.19 (C1), 129.42 (C2), 129.34 (C3), 126.85 (C4), 129.34 (C5), 129.42 (C6), 105.30 (C1'), 165.81 (C2'), 95.75 (C3'), 166.14 (C4'), 95.75 (C5'), 165.81 (C6'), 46.90 (Cα), 32.21 (Cβ), 206.04 (C=O).

*Dihydroflavokawain C* (**4**). Crystalline needles, UV λmax (MeOH) 232, 285 nm. HR-ESI-MS *m/z*: [M+H]^+ ^ = 303.1232 for C_17_H_18_O_5_]. ^1^H-NMR (CDCl_3_) δ 7.09 (2H, d, *J* = 8.4 Hz, H2 and H6), 6.76 (2H, dd, *J* = 8.4 Hz, H3 and H5), 6.07 (1H, d, *J* = 8.4 Hz, H3'), 5.92 (1H, d, *J* = 8.4 Hz, H5'), 3.81 (3H, s, OCH_3_-4'), 3.83 (3H, s, OCH_3_-5'), 14.0 (1H, s, OH-2'), 3.27 (2H, dd, *J* = 7.4, 8.4 Hz, Hα), 2.92 (2H, dd, *J* = 7.4, 8.4 Hz, Hβ). ^13^C-NMR (CDCl_3_): δ = 133.8 (C1), 129.5 (C2), 115.2 (C3), 153.8 (C4), 115.2 (C5), 129.5 (C6), 105.7 (C1'), 162.7 (C2'), 90.8 (C3'), 166.0 (C4'), 93.7 (C5'), 167.6 (C6'), 55.6 (OMe-2'), 55.5 (OMe-4'), 45.9 (Cα), 29.8 (Cβ), 204.7 (C=O).

### 3.5. Biological Assays

Assays were performed according to the methodology recommended by the WHO [17,18], and experimental procedures were employed according to accepted principles of animal welfare in experimental science.

Adults and egg masses of *B. glabrata* (Say, 1818) were obtained from a Belo Horizonte population (MG, Brazil) and reared under laboratory conditions for several years, with fresh lettuce *ad libitum* for maintenance and a balanced ration during the assay.

In all assays, both positive and negative controls were used to examine the susceptibility of the organisms under the assay conditions. The commercially available molluscicide niclosamide was used in the positive control group; the negative control group received dechlorinated tap water containing 1% DMSO.

### 3.6. Molluscicidal Activity

Snails with 10–18 mm of shell diameter were exposed to *P. diospyrifolium* extract and isolated compounds (Table 1, Supplementary Table S1) at concentrations less than 40 mg/mL for 24 h at 24 °C ± 2 °C. After exposure, the snails were washed, observed daily for 7 days, and the death rate was recorded [19]. The LC_90_ and LC_50_ values were then determined from the death rate data. Ten animals were used per concentration and experiments were repeated three times.

### 3.7. Ovicidal Activity

Plastic sheets served as the substrate for oviposition, and small circles with one egg mass attached were excised. Five egg masses at the blastula, gastrula, trocophore and veliger stages [20] were exposed to isolated compounds at concentrations less than 20 mg/mL for 24 h to determine the LC_90_ and LC_50_ values. The number of snail embryos to each concentration is indicated in Supplementary Table S1. Following the exposure, the egg masses were washed with dechlorinated water and Petri dishes containing egg masses were kept within climatic chambers with a controlled temperature (25 °C ± 1 °C). All egg masses were examined daily for 7 days under a stereomicroscope. Embryos were considered as dead whenever disintegrating embryonic forms were noted within the egg and/or at later developmental stages when no heartbeats were detected. Assays were repeated three times with approximately 100 embryos for each concentration.

### 3.8. Docking

The structure of the RXR-like protein (retinoid X receptor) (PDB code: 1XIU) was downloaded from Protein Data Bank [21]. The ligands were drawn using the Marvin Sketch v. 6.1.4 [22], and the three dimensional structures were generated using the software Standardizer v 6.1.4 [23]. Compounds **1**–**6** were submitted to molecular docking using the Molegro Virtual Docker v. 6.0.1 (MVD) [24,25]. All the water molecules were deleted from enzyme structure, the enzyme and ligands were prepared using default parameter settings on the same software. The active site was the same as that containing the crystallized ligand (retinoic acid). The coordinates of the constraint were: x = 33.87; y = 38; z = 90.28. Moldock score [GRID] algorithm was used as the score function and the search algorithm was Moldock SE [24]. Rank and re rank were used to select the poses with lowest Moldock energies.

### 3.9. Molecular Interaction Fields

The structures modeled as described above were used as the initial structures to generate molecular descriptors employing the VolSurf+ v 1.0.7 program [26,27]. The descriptors were generated using the following probes: N1 (amide nitrogen—hydrogen bond donor probe), O (carbonyl oxygen—hydrogen bond acceptor probe), OH2 (water probe), and DRY (hydrophobic probe), totalizing 57 descriptors [12]. PCA (Principal Component Analysis) was applied to the investigated set using the same software [24].

## 4. Conclusions

The evaluated compounds were active against *Biomphalaria glabrata*, and docking simulations described the interaction of all of the compounds in the RXR-like protein’s hydrophobic retinoic acid binding pocket. Molecular interaction fields determined the physical-chemical features that explained the highest activity of compounds **2** and **3**. Compared to niclosamide (LC_50_ 0.05 μg/mL) [19], the activity of the isolated compounds displayed moderate molluscicidal activity. However, considering the limitations of niclosamide, the compounds investigated in this study are promising molluscicides derived from botanical sources.

## Supplementary Materials

Supplementary materials can be accessed at: http://www.mdpi.com/1420-3049/19/4/5205/s1.
