# Supplementary material for: A Benzoic Acid Derivative and Flavokawains from Piper species as Schistosomiasis Vector Controls
Source: Molecules. 2014 Apr 23;19(4):5205–18. doi: 10.3390/molecules19045205 (PMC6271750; doi:10.3390/molecules19045205)

Supplementary Files

Table S1. Molluscicide activity of the compounds **1**, **2**, **3**, **4**, **5** and **6** in different developmental stages.

**Compounds**	**Adults**		**Embryos**			
	**Concentration (µg/mL)**	**Dead (%)**	**Developmental Stage**	**Concentration (µg/mL)**	**n °embryos**	**Dead (%)**
**flavokawain A (1)**	0 *	2 (6.6)	Blastula	0 *	107	0
	5	1 (3.3)		20	129	0
	10	5 (33.3)	Gastrula	0 *	102	0
	20	12 (66.6)		20	105	0
	30	26 (86.6)	Trocophore	0 *	115	0
	40	30 (100)		20	142	0
			Veliger	0 *	127	0
				20	117	0
**2',4',6'-trihydroxy dihydrochalcone [1] (3)**	0 *	1	Blastula	0 *	119	0
	1	0		5	102	0
	2	2 (20)		10	116	61 (52.5)
	4	3 (30)		15	134	113 (84.3)
	6	6 (60)		20	162	162 (100)
	8	9 (90)	Gastrula	0 *	110	0
	10	10 (100)		5	112	0
				10	124	57 (45.9)
				15	119	107 (89.9)
				20	131	131 (100)
			Trocophore	0 *	107	0
				5	128	0
				10	148	62 (41.8)
				15	98	80 (81.6)
				20	171	171 (100)
			Veliger	0 *	109	0
				5	137	0
				10	114	35(30.7)
				15	144	121 (84)
				20	124	124 (100)
**dihidroflavokawain C [1] (4)**	0 *	0	Blastula	0 *	98	0
	20	5 (50)		20	102	0
			Gastrula	0 *	86	0
				20	115	0
			Trocophore	0 *	98	0
				20	113	0
			Veliger	0 *	98	0
				20	122	0
**4-hydroxy-3-(3,7,11-trimetildodeca-2,6,10-trienyl) benzoic acid (2)**	0 *	2	Blastula	0 *	113	0
	2	3 (10)		20	94	0
	4	9 (30)	Gastrula	0 *	113	0
	6	9 (30)		20	114	0
	8	17 (56.6)	Trocophore	0 *	99	0
	10	23 (76.6)		20	91	0
	12	30 (100)	Veliger	0 *	111	0
				20	117	0

Table S1. *Cont.*

**Compounds**	**Adults**		**Embryos**			
	**Concentration (µg/mL)**	**Dead (%)**	**Developmental Stage**	**Concentration (µg/mL)**	**n °embryos**	**Dead (%)**
**Hydroquinone (6)**	0 *	0	Blastula	0 *	302	0
	0.5	0		0.25	375	0
	1.5	7 (23.3)		0.5	369	39 (10.5)
	3	14 (46.6)		1.0	386	138 (35.7)
	4.5	21 (70)		1.5	406	344 (84.7)
	6	27 (90)		2.0	442	442 (100)
	7.5	28 (93.3)	Gastrula	0 *	302	0
	9	30 (100)		0.5	403	3 (0.7)
				1.0	337	47 (13.9)
				2.0	340	171 (50.2)
				3.0	369	330 (89.4)
				4.0	440	440 (100)
			Trocophore	0 *	387	0
				0.5	400	0
				1.0	408	86 (21)
				2.0	460	230 (50)
				4.0	373	212 (56.8)
				6.0	440	379 (86.1)
				8.0	368	368 (100)
			Veliger	0 *	301	0
				1.0	385	0
				2.0	433	129 (29.7)
				4.0	398	294 (73.8)
				6.0	351	289 (82.3)
				8.0	407	407 (100)
***p*-hydroxybenzoic acid [1] (5)**	0 *	0	Blastula	0 *	114	0
	20	0		20	132	0
			Gastrula	0 *	115	0
				20	155	0
			Trocophore	0 *	102	0
				20	114	0
			Veliger	0 *	115	0
				20	102	0

N = 30 snails for the adult stage and 15 egg masses per concentration; [1] n = 10 snails for the adult stage and 5 egg mass per concentrations; * 0 = negative control—DMSO 1%. Values were obtained at the end of the 7th day of observation.

Figure S1. Binding conformations of retinoic acid and compound **2**. Blue surface represents hydrophobic favourable areas, the red surface represents hydrophilic favourable areas. Blue dash lines represent hydrogen bond interactions.

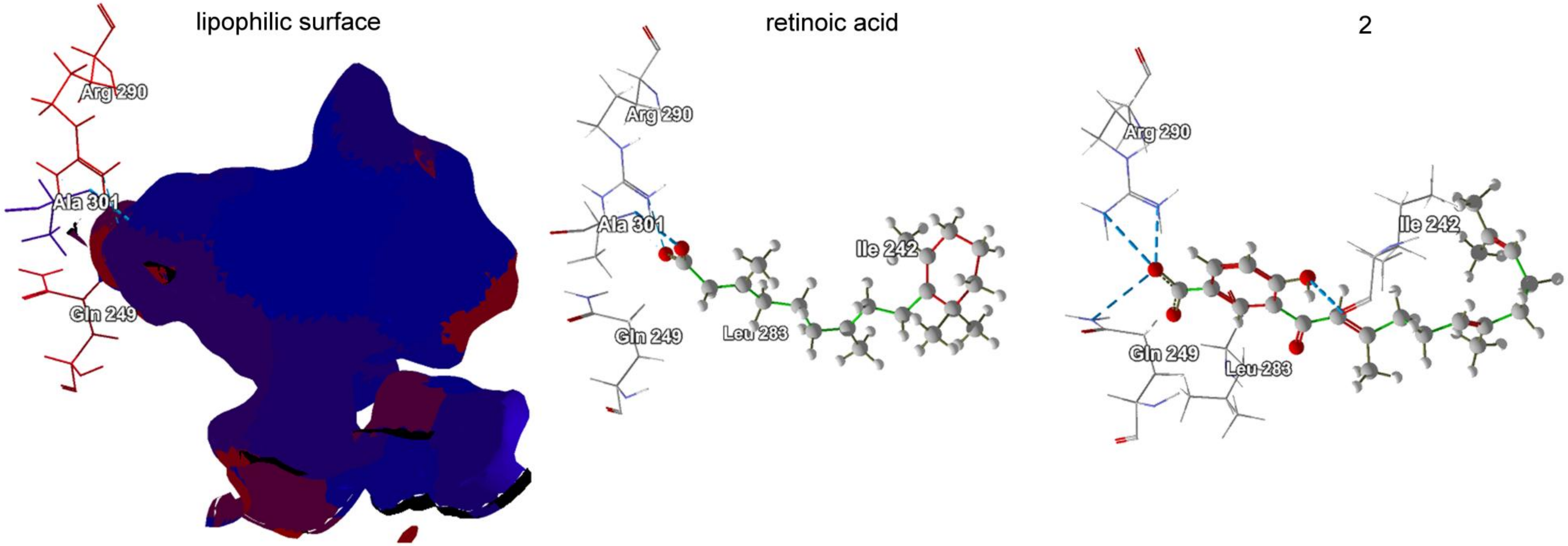

Figure S2. Maps of interaction with the probes water (light blue), amide nitrogen (blue), carboxylic oxygen (red) and DRY (lipophilic - green) probes for the set studied.

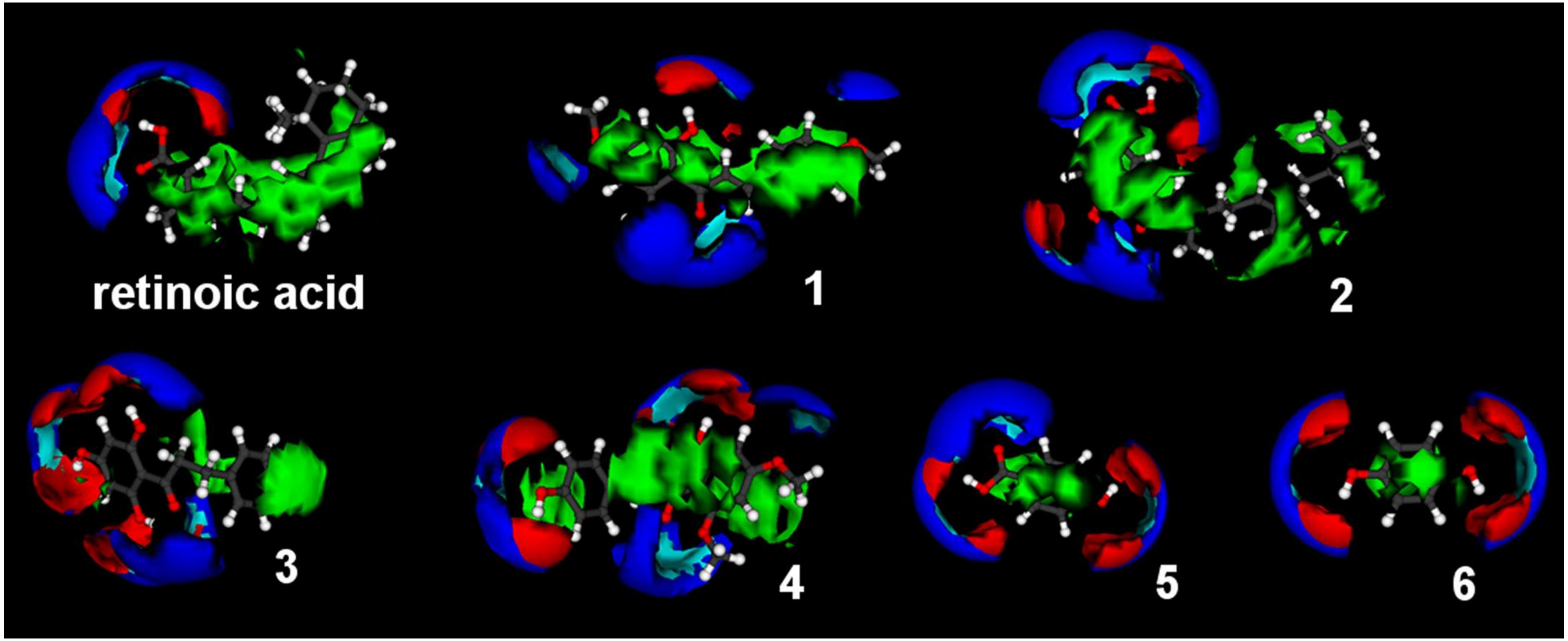

Supplement: Supplementary file 1 [file molecules-19-05205-s001.pdf]
